# Blood Group Testing

**DOI:** 10.3389/fmed.2022.827619

**Published:** 2022-02-11

**Authors:** Hong-Yang Li, Kai Guo

**Affiliations:** ^1^Department of Blood Transfusion, China-Japan Union Hospital of Jilin University, Changchun, China; ^2^Department of Transfusion Medicine, Beijing Children's Hospital, Capital Medical University, National Center for Children's Health, Beijing, China; ^3^National Center for Clinical Laboratories, Institute of Geriatric Medicine, Chinese Academy of Medical Sciences, Beijing Hospital/National Center of Gerontology, Beijing, China; ^4^Graduate School of Peking Union Medical College, Chinese Academy of Medical Sciences, Beijing, China

**Keywords:** red blood cell, blood group typing, blood group testing, blood group antigen, ABO blood group system, RH blood group system

## Abstract

Red blood cell (RBC) transfusion is one of the most frequently performed clinical procedures and therapies to improve tissue oxygen delivery in hospitalized patients worldwide. Generally, the cross-match is the mandatory test in place to meet the clinical needs of RBC transfusion by examining donor-recipient compatibility with antigens and antibodies of blood groups. Blood groups are usually an individual's combination of antigens on the surface of RBCs, typically of the ABO blood group system and the RH blood group system. Accurate and reliable blood group typing is critical before blood transfusion. Serological testing is the routine method for blood group typing based on hemagglutination reactions with RBC antigens against specific antibodies. Nevertheless, emerging technologies for blood group testing may be alternative and supplemental approaches when serological methods cannot determine blood groups. Moreover, some new technologies, such as the evolving applications of blood group genotyping, can precisely identify variant antigens for clinical significance. Therefore, this review mainly presents a clinical overview and perspective of emerging technologies in blood group testing based on the literature. Collectively, this may highlight the most promising strategies and promote blood group typing development to ensure blood transfusion safety.

**Graphical Abstract:**
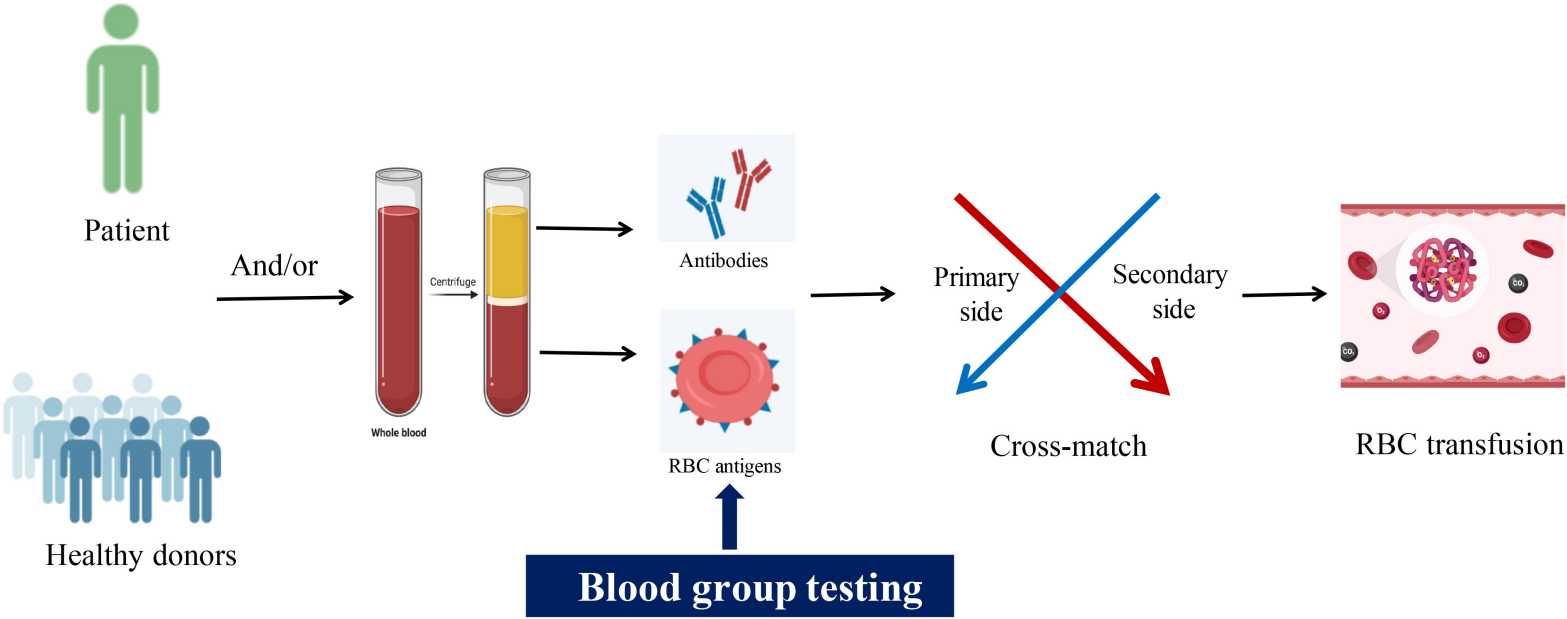
Emerging technologies for blood group typing. Image created with BioRender.com.

## Introduction

Blood group antigens in human red blood cells (RBC) can evoke immune antibodies capable of causing immune-mediated hemolysis. That is, blood group antigen testing is essential to save the lives of patients undergoing blood transfusion. Generally, a cross-match test is necessary to observe and assess the compatibility between donor and recipient blood groups before blood transfusion. Currently, there are 43 blood group systems containing 345 antigens for human RBCs recognized by the International Society of Blood Transfusion (ISBT, available from: http://www.isbtweb.org, accessed 12/28/2021) Working Party, which was established in 1980 in England, works in conjunction with the International Blood Group Reference Laboratory to develop a professional numerical terminology based on blood group genetics and plays a key role in ensuring patient safety in blood transfusion. A blood group system comprises inherited antigens by a single gene or a cluster of two or more closely linked homologous genes and is defined serologically by a specific antibody. The 43 blood group systems are genetically determined by 48 genes. A blood group system-associated number and symbol was terminology designated and maintained by the ISBT Working Party for Red Cell Immunogenetics and Blood Group Terminology, for example, “001” and “ABO” for the ABO blood group system, “004” and “RH” for the RH blood group system (https://www.isbtweb.org/fileadmin/user_upload/Table_of_blood_group_systems_v10.0_30-JUN-2021_with_LRG_and_revised_antigens.pdf).

Patients who are awaiting transfusion, pregnant women, blood donors, etc., needed to be routinely tested for the ABO and RH(D) antigens, which are the essential antigens for ensuring patient transfusion safety. Blood transfusions may lead to hemolytic transfusion reactions without ABO and RH(D) compatibility testing. Testing for other blood group antigens, such as MNS, Lewis, Kell, Duffy, and Kidd, is sometimes necessary for patients who harbor or are significantly likely to develop antibodies against these antigens (1). Correct blood group typing is critical for ensuring blood transfusion safety and is also essential for several clinical tests and research settings. Considerable advances have been made in recent years in identifying different blood groups, and novel techniques have been developed for blood group testing. In this review, we have summarized the current blood group testing methods and discussed the clinical applications of novel typing techniques.

## ABO Blood Group System

**Figure 1:**
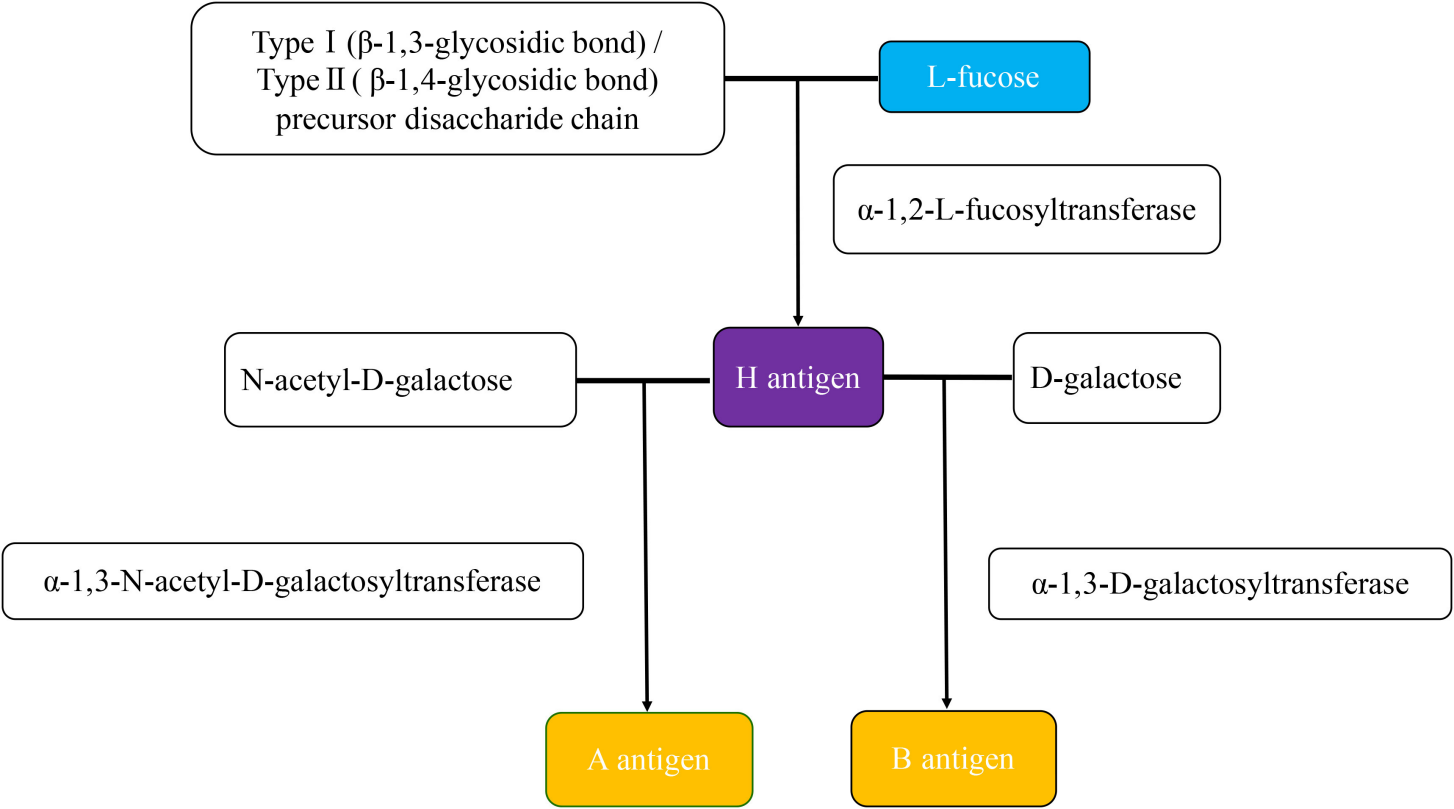
The production of ABH antigens. The H antigen is produced when an α-1,2-L-fucosyltransferase catalyzes an L-fucose transfer to the precursor disaccharide chain of type 1 (soluble) or type 2 (cell-bound). The difference between type 1 and type 2 precursor disaccharide chains is that the residue terminal disaccharides are linked to different glycosidic chains. Type 1 is the β-1,3-glycosidic bond, and type 2 is the β-1,4-glycosidic bond. The H antigen is the precursor of both the A and B antigens. The N-acetyl-D-galactose for A antigen, D-galactose for the B antigen. The actions of α-1,3-N-acetyl-D-galactosyltransferase and α-1,3-D-galactosyltransferase are responsible for forming the A and B antigens, respectively.

The ABO blood group was first discovered in 1900 by Karl Landsteiner showed experimentally by cross-testing RBCs and sera, and is classified into type A, type B, type AB, and type O based on five glycoprotein antigens—A, B, AB, A1, and H—that are expressed on the surface of RBCs later. In addition, ABH oligomers are also present on the surface of other cells and in bodily fluids or secretions. The glycosyltransferases of A, B, and H antigens transfer different monosaccharides to the non-reducing terminals of glycoproteins and glycolipid-specific glycans and produce the different terminal glycosyls (blood group epitopes). The α-1,2-fucose transferase (FUT) plays a crucial role in creating H antigen. The H antigen on RBCs and in secretion is encoded by the H (FUT1) gene and Se (FUT2), respectively. The A and B antigens are carbohydrate antigens built upon the H antigen. The N-acetyl-D-galactose is added at the end of the H antigen in the action of α-1,3-N-acetyl-D-galactosyltransferase, creating the A antigen. The action of α-1,3-D-galactosyltransferase on the H antigen adds D-galactose, producing the B antigen. Both transferases in the AB blood group generate A and B antigens. The resulting blood group is O when neither transferase is present. See details in Figure 1 (1–4).

The lack of an ABO antigen increases the titer of antibodies against that antigen in the serum. For instance, individuals with A blood group contain the A antigen and anti-B antibodies. In addition, most individuals with the type A blood group are of type A1 and harbor both A and A1 antigens, whereas the subtype A2 contains only antigen A and is rare. Likewise, blood type B consists of the B antigen and anti-A antibodies. The AB blood type contains both antigens and neither antibodies. O blood type lacks either antigen and has anti-A and anti-B antibodies (5). The ABO blood group antibodies are naturally occurring and mostly of the immunoglobulin IgM class, produced without antigen stimulation or during the classical adaptive immune response, which remains controversial (6, 7). Arend P (8) suggested that allogeneic blood type antibodies are initially determined by an ancestral gene that “spontaneously” produces B1 lymphocytes. Following transfusion of an incompatible blood type, the A and B antigens and the corresponding antibodies form complexes and trigger a robust immune response, resulting in a fatal hemolytic reaction.

## RH Blood Group System

The RH blood group system is second only to the ABO blood group system for determining blood transfusion safety. Currently, it includes 56 antigens, of which the D, C, c, E, and e antigens are most relevant to blood transfusion, especially the D antigen. RH blood typing determines the presence or absence of D antigen on RBCs using an anti-D reagent. D-positive is RH-positive, and D-negative is RH-negative. Weak or partial D antigens may present in some individuals, such as newborns with D-negative mothers, due to reduced antigenic sites or the loss of extracellular D epitopes (9–13). In such cases, a direct agglutination test with the anti-D reagent may result in false-negative results, and agglutination can only be observed with an anti-human immunoglobulin reagent.

## Blood Group Testing Methods

Generally, blood typing can be classified as forward and reverse typing based on traditional serological testing (14, 15). Specifically, forward typing involves the detection of A and B antigens on the basis of RBC agglutination by the specific antiserum and the intensity of agglutination, whereas reverse typing is used to identify the presence or absence of anti-A, anti-B, and anti-AB antibodies in plasma or serum. The titer of the ABO antibodies is also of great significance in clinical blood transfusion and is commonly measured by the test tube method, the microcolumn gel card method, etc. However, each of these serological methods is based on hemagglutination and is therefore affected by subjective factors, making standardization difficult and decreasing the accuracy. It is always mandatory to test the ABO type prior to blood transfusion. Nevertheless, there is no specific antiserum or technology that can guarantee the detection of all rare, weak, or variant antigens. Moreover, certain diseases, such as acute myeloid leukemia, can decrease the level of blood group antigens (16). The conventional and novel methods for blood group testing have been described and discussed in the following sections.

### Slide Testing

The slide method is a simple and rapid (5–10 min) method for blood typing that uses small amounts of reagents and is routinely used for emergency blood group testing (17). However, the sensitivity of this method is low and can be easily affected by multiple factors, thus making standardization difficult. In addition, weakly expressed or rare antigens cannot be easily detected by the slide method. Low-titers of anti-A or anti-B antibodies often lead to false-negative results, which increases the risk of mistyping before blood transfusion (18).

### Tube Testing

The test tube method is a more sensitive and faster alternative to the slide method that requires lower amounts of reagents and is suitable for emergency and initial blood group typing. The RBCs and antibodies mixture is centrifuged, and the result is easily interpreted by visual examination. If the RBC sediment is undisturbed after centrifugation for a few minutes and gently shaking the test tube, it is considered complete agglutination. On the other hand, uniform distribution suspension of the RBCs indicates non-agglutination. In the event of a lack of any obvious agglutination, the RBC suspension is dropped on a glass slide, and the degree of agglutination is observed under a microscope. The interpretation of these results lacks objectivity and a unified standard. In addition, this method cannot be used to accurately determine the blood group in infants (reverse typing), leukemia patients with weak antigens, and patients with low antibody titers, etc. It is imperative to clean test tubes, be precise with the centrifugation time and speed, and observe the results under ambient conditions to assure the accuracy of this testing (19). Tube testing is also challenging to automate. Despite its disadvantages, the test-tube method is still a prevalent technique that has been used for blood type identification and validation.

### Microplate Agglutination Method

The microplate technology uses automated platforms to detect serum antibodies and RBC surface antigens. The reactants are centrifuged and incubated in microplates, and the ABO/RH(D) blood type is read through an automated system (20, 21). It has the advantages of speed, low reagent consumption, and high-throughput analysis. The U-shaped plate method is fully automated and involves the addition of plasma and RBCs, centrifugation, incubation with constant shaking, and final observation and interpretation (22). The appearance of RBC clots or fine sand in the U-shaped micropores indicates agglutination. The U-shaped plate can detect multiple specimens simultaneously, which saves both time and labor. However, since the U-shaped plate needs to be clean and transparent to prevent fibrin from affecting RBC agglutination, the process must be strictly standardized to ensure the accuracy of test results. Studies show that the accuracy of blood typing using *U*-shaped plates is as high as 99.6% (23), which is comparable to that of the test tube method.

Generally, the ladder microplate is a 96/120-well trapezoid or V-shaped plate with a stepped hole wall, and the width of each step is approximately equal to whereas the height exceeds the diameter of one RBC. Following the antigen-antibody reaction, the RBCs aggregate through covalent bonds and van der Waals forces and adhere uniformly to the wall of the hole. In the event of no antigen-antibody reaction, the RBCs sink along the ladder to the bottom of the hole into a solid dot. The addition of samples is automated through an enzyme immunoassay instrument, and the results are detected using digital imaging technology (24). Both visual and text results are automatically transmitted to the laboratory management system, which is convenient to retrieve and avoids manual recording errors. The trapezoidal microplate can be used repeatedly to simultaneously analyze numerous samples, which significantly reduces analysis time and labor.

### Microcolumn Gel Method

The column gel agglutination technology was introduced by Lapierre et al. (25) in 1990. Both manual and automated testing are available. The automated microcolumn gel method is widely used for ABO and RH blood typing. It is the relatively standard method for RBC typing in clinical laboratories and blood banks. This technology is a straightforward, convenient, and quick method that uses small amounts of reagents and has high sensitivity, accuracy, and reproducibility, which dramatically reduces identification errors caused by human factors. The microcolumn gel testing card has forward and reverse typing, just like the test tube method for blood type testing. Additionally, utilizing glass beads instead of gel material helps further reduce analysis time, with quicker centrifugation speeds being reached (17). Except for ABO and RH antigens, other RBC phenotypes will also be tested with updated microcolumn gel cards and instruments.

### Novel Paper-Based Testing

A novel paper-based test can simultaneously determine forward and reverse ABO and RH blood groups within 10 min based on the paper-based analytical device fabricated by using a combination of wax printing and wax dipping technique in real blood sample analysis (26). Compared with slide testing, the accuracy of this assay for blood groups A, B, O, and AB ranges from 85 to 96%. Six parallel channels are printed with wax on the filter paper in a paper-based ABO blood group analysis (27). The forward and reverse ABO blood group typing were strongly correlated with the slide and tube testing (27). Compared to ordinary paper, nitrocellulose-based paper can fix agglutinated blood cells using less sample volume and exhibits higher accuracy and reproducibility (28). Due to the higher degree of separation between agglutinated and non-agglutinated RBCs on the nitrocellulose matrix, the color changes are more significant. After the blood sample is mixed with the blood group antibody, the change in the bloodstain area over time is measured. A rapid paper-based blood group testing method from droplet wicking was found to have a high sensitivity, which was sensitive to antibody dilution between 1:2 and 1:8. It can determine ABO groups within 10 seconds and RH(D) for 20 seconds (29). The use of low-density and thick paper was more sensitive to detection (29).

Additionally, bioactive paper for blood group typing has been established by observing RBCs' haemagglutination (30–33), such as Kleenex paper towels (34, 35). The low cost, quickness, portability, and ease of paper-based testing holds considerable potential for point-of-care blood group typing with naked-eye readouts (27, 29, 36). The paper-based analytical device may be further developed for future applications in blood group testing.

### Dye-Assisted Paper-Based Detection

Several clinical situations, such as excessive blood loss, require rapid bedside detection of the patient's blood type (37–40). The traditional detection method requires 5–45 min to determine the blood type, and weak agglutination identification remains challenging. In general, routine paper testing techniques may encounter inherent environmental challenges and low precision during the readout process. A novel method was developed for rapid and reliable blood typing using linter paper as the matrix and the dye bromocresol green that changes color in the presence of different blood components with flipping identification with a prompt error-discrimination platform and the color correction algorithm (41). As a result, the color change of the indicator dye can be used to determine the blood group. Cotton lint paper has larger fiber space, smaller pores, and greater blood-absorbing capacity compared to 3MM chromatography paper, making it more suitable for blood flow and stable antibody coating. Once the blood sample is dropped on the paper, it penetrates the deeper layer and reacts with the coated antibody. Depending on whether or not agglutination occurs, bromocresol green undergoes a specific color change in the reaction zone. The bromocresol green paper strip can accurately detect the ABO/RH blood type within 30 s and determine 16 rare blood types within 3 min.

In one study, this method exhibited similar accuracy and reproducibility as the classical column assay when tested on 450 blood samples (41). Based on the same principle, another group developed bromocresol green dye and antibody-coated paper strips, which typed 3,550 human blood samples with comparable accuracy to the classic gel-card analysis (42). To summarize, the paper-based detection matrix is simple, portable, fast, and convenient (43); combined dye assist may further benefit blood typing.

### Microfluidic Testing

Rapid and accurate blood group typing is essential in emergency scenarios for safe blood transfusion. A digital microfluidic droplet agglutination assessment detection method was recently developed for rapid blood group typing prior to emergency transfusion (44). It depends on digital microfluidics that manipulates discrete nano- to microscale droplets on an open electrode array by increasing the electric potential. The digital microfluidics-based detection system includes a DropBot droplet control system, reconstituted pumps for freeze-drying analysis reagents, cameras, laptops, etc. (44–47). The specimens can be collected, processed, and measured using an automated system and simple portable instruments that can operate in remote and difficult environments with limited access to centralized laboratories (48). Using this method, the blood type of 60 blood samples was tested and validated with 100% accuracy (44). The detection system can also perform compatibility detection with the donor and hematocrit analysis (44). Since the entire process takes <6 min, it is highly suitable for emergency trauma patients (44).

Another microfluidic device was developed to type the ABO/RH(D) blood groups and identify the weak antigens (49, 50). The process requires only 1μl of blood collected by pricking the fingertip, which is then mixed with 10 μl of PBS in a reaction tank (49). The sample passes through a four-layer microfluidics chip pre-loaded with anti-A, anti-B, and anti-D antibodies (0.5 μl). The agglutinated RBCs accumulate in the channel, and the formation of a distinct red line within 2 min is a positive result. The length of the red line is directly proportional to the agglutination intensity. A microfluidic thread-based analytical device was also used to accurately identify six weak A subgroups and 89 normal ABO groups (50). This device can use a manual pump system that does not need to rely on electricity and therefore can be used outdoors, at home, or in an emergency vehicle (51).

Additionally, in one study, ABO and RH(D) blood groups were detected using an antibody microarray on the poly (methylmethacrylate) surface coupled with the microfluidic system (52). The Poly (methylmethacrylate) microarray chip showed good reproducibility, accuracy, and precision. The platform is simple to fabricate for mass manufacturing (53, 54). The microfluidic system allows very fast visual detection with a minuscule amount of the sample, which also lowers the cost.

### Waveguide-Mode (WM) Sensor Testing

A waveguide-mode (WM) sensor can detect particles and molecules on the sensor chip using electric field enhancement with waveguide modes (55, 56). A WM sensor is a portable, instantaneous blood group testing device with a portable battery that does not require an electricity supply. In emergency scenarios, WM sensors can determine ABO and RH(D) blood types by utilizing hemagglutination detection within 3.5 min (57). Sensor chips made of glass are versatile, and the wavelength of incident light is easy to control (57). Microfluidic multichannel WM sensor chips that are mechanically stable are available for automated, simultaneous blood group testing (58, 59). A WM sensor equipped with a microfluidic channel can determine forward ABO typing in <1 min (58).

### Erythrocyte-Magnetized Technology

Erythrocyte-magnetized technology (EMT) is a fully automated blood typing technology for ABO and RH phenotyping and antibody on the basis of the magnetization of RBCs (60–62). Magnetized erythrocytes for the rapid transfer of antigens or antibodies through simple magnets benefit automated diagnostic equipment (62). The simplicity of automation made it highly reliable and ideal for medium-to-extensive facilities and saved a lot of staffing; the high level of security and total traceability perfectly respond to excellent clinical laboratory practice requirements (62).

### Protein Chip Testing

Generally, a protein chip or protein microarray is a type of proteomic technology that involves the immobilization of hundreds of distinct proteins (63, 64). Following the antigen-antibody reaction, secondary antibodies labeled with fluorescence tracers or magnetic beads that are specific for different epitopes of the target antibody are added (65). Magnetic beads are used more frequently due to lower costs. In addition to high sensitivity and specificity akin to enzyme-linked immunosorbent assays, the protein chip also has the advantages of high throughput, high parallelism, and miniaturization of the chip technology (66). A protein chip consists of a substrate coated with blood group antigens or antibodies that can detect the corresponding antibodies/antigens in RBC fragments or serum samples (67, 68). A visual protein chip for ABO blood group testing was successfully fabricated based on magnetic beads (67). The results of the protein chip and test tube methods are consistent, and the former has higher sensitivity and specificity. Additionally, in one study, a multiplexed bead-based immunoassay was developed utilizing reproducible and robust RBC blood group antigen arrays constructed using fragmented RBC membranes (68). Compared to the gel microcolumn assay, this approach showed tremendous promise in screening blood group antibodies with higher sensitivity and quantitatively.

### Surface Plasmon Resonance Testing

Surface plasmon resonance **(**SPR) is a sensitive technique and is a label-free method for tracking molecular interactions in real-time (69). In some applications, the sensitivity and versatility of the label-free, real-time technique outperform traditional methods like enzyme-linked immunosorbent assays or fluorescence (70). Generally, RBC antigen-specific IgM or IgG antibodies immobilized on the surface of SPR can be used to identify blood groups (71, 72). The intensity of agglutination depends on RBC adhesion and wall shear stress (73). The adhered RBCs move at different average cell velocities under the shear flow, which produces resistance. Higher average cell velocity decreases the resistance of antigen and antibody binding and lowers agglutination intensity. The antigen density of A_1_ and B RBCs from that of A_1_B RBCs and can be resolved using this approach (73).

SPR imaging has the potential to be used as a high-throughput bioanalyzer in protein analysis (74–76). Using SPR imaging as a high-throughput technique for ABO and RH blood group typing is a promising strategy (77, 78). Blood samples were correctly used for ABO blood typing within 12 min by the SPR imaging and were consistent with the standard agglutination methods (72). SPR biodiagnostics using the sensor chip can quantitatively detect blood groups, which could benefit from detecting weaker variants (79). The D-antigen was validated as an example for quantitative blood grouping (79). Using the long-range SPR biosensor in a long-range SPR blood group typing, the results were in full accordance with those of the agglutination test (70). In a word, this method has high accuracy and sensitivity and requires a small sample quantity and little time. However, SPR relies on expensive equipment and consumables and may not be practical for routine blood group testing.

### Flow Cytometry Testing

A flow cytometry-based method has been established to detect the human ABO group and D type. It has higher specificity, sensitivity, and reproducibility compared to traditional hemagglutination methods and can easily detect immunoglobulin subtypes in addition to quantifying the antibodies (80–84). Flow cytometry is capable of detecting tiny subpopulations as an alternative and supplemental approach when blood groups cannot be detected by serology and results can be delivered in less than an hour (85). Flow cytometry identifies the recipient and donor blood using the transferrin receptor and the fluorescent stain Thiazole Orange RNA as a maturity index of reticulocytes (86). Therefore, flow cytometry for blood typing on immature reticulocytes can be achieved, particularly in the recipient population (85).

Blood groups can also be typed using dual-color CdTe quantum dots to quantify antigen expression on the RBC surface by flow cytometric analysis (87). Quantum dots were conjugated to anti-A/anti-B and the anti-H (Ulexeuropaeus I) lectin to study A_1_, B, A_1_B, O, A_2_ RBCs, and weak donors. With high sensibility and specificity, this approach can be used as a complementary and versatile analysis to better understand a variety of RBC antigens (87). Flow cytometry testing is a multi-parametric, relatively easy, and not expensive approach. On the other hand, ~1 h may not be suitable for trauma care settings.

### Genotyping

Blood group genotyping methods can be divided into low-throughput, medium-throughput, and high-throughput techniques (88, 89). Different throughput blood group genotyping methods are shown in Table 1. Low-throughput techniques include polymerase chain reaction-restriction fragment length polymorphism (PCR-RFLP), PCR-allele specific primer (PCR-ASP), PCR-sequence specific oligonucleotide (PCR-SSO), PCR-single strand conformation polymorphism (PCR-SSCP), PCR-sequence specific primers (PCR-SSP), etc. (90–92), which are mostly based on the detection of known single nucleotide polymorphism (SNP) (89, 93, 94), and therefore may miss unknown gene mutations. Although PCR-SSP can detect the weak antigens and subtypes of the ABO system, it can only analyze a few key sites in the ABO gene. Medium-throughput techniques include real-time PCR, Sanger DNA sequencing, and pyrosequencing, etc. Real-time fluorescent PCR was used to genotype the Diego (95) and Duffy (96) blood groups, and Atamaniuk et al. (97) used the TaqMan probe method to predict the fetal RH(D) blood type in 2009.

**Table 1:** Blood group genotyping methods.

**Groups**	**Test methods**
Low-throughput	PCR-RFLP, PCR-ASP, PCR-SSO, PCR-SSCP, PCR-SSP
Medium-throughput	Real-time PCR, DRAM, Sanger DNA sequencing, pyrosequencing
Medium- to high-throughput/ High-throughput	Mini sequencing, MALDI-TOF-MS, NGS, WGS
Semi-automatic platforms	SNP stream, snapshot assay, open array, floating array, multiple ligation-dependent probe amplification analysis

Medium- to high-throughput/High-throughput typing techniques include mini sequencing or the snapshot assay, matrix-assisted laser desorption/ionization time-of-flight mass spectrometry (MALDI-TOF-MS) (98–100), and next-generation sequencing (NGS), etc. (101–103). Di Cristofaro et al. (104) successfully analyzed multiple blood group systems by microsequencing. In addition, NGS can identify blood group antigens on the basis of specific gene sequences and detect all polymorphisms, including null alleles, new mutations, and complex gene rearrangements (105–109). Therefore, NGS is a highly suitable approach for identifying rare and mutant blood types with no known specific antibodies (110, 111). The microarray technology (112) can simultaneously identify numerous SNPs and alleles of different blood group systems, thereby allowing genotyping of multiple blood group systems. In addition, the microarray technology generally involves high throughput automated and can detect and analyze a large number of blood group DNA polymorphisms (112, 113). However, the sensitivity of the microarray chip decreases after repeated use, sample preparation, and labeling are technically demanding, and it can only detect known alleles. Semi-automatic platforms based on microarray assays have also been developed for blood group genotyping (1), including SNP stream (114, 115), snapshot (116, 117), open array (118, 119), suspension array (120), and multiplex ligation-dependent probe amplification analysis (121).

However, the aforementioned techniques generally require DNA purification, which is usually the most speed-limiting and labor-intensive step. On the other hand, direct, real-time allele-specific PCR and melting curve analysis (DRAM) is a fast and reliable one-step blood group genotyping technique that does not require DNA preparation (122). It uses a special buffer for direct PCR, rapid RBC lysis buffer, white blood cell DNA template, allele-specific primers, and DNA-binding fluorescent dyes (EvaGreen; Biotium) for discriminating ABO alleles. The PCR reaction process is carried out in a closed system, reducing manpower and material resources and the risk of contamination. Studies showed that DRAM measurement was 100% consistent with the ABO genotyping results of PCR-RFLP, PCR direct sequencing, and serological typing results. Compared to traditional ABO genotyping utilizing allele-specific PCR with purified DNA and agarose gel electrophoresis, DRAM reduces manual procedures to hands-on time from ~40 to 12 min. The total time required for the DRAM assay is around 274 min.

Additionally, NGS has the capacity to sequence the whole genome (WGS), but is neither cost-efficient nor practical for clinical transfusion laboratories. Nevertheless, WGS is increasingly performed to discover unknown or undiscovered allelic polymorphisms, blood group antigen phenotypes, and associations between genes and certain chronic diseases (105). In one study (123), the whole genome data of 79 individuals with nine red blood cell antigen systems was analyzed, and the consistency of blood group polymorphism was 93%. A total of 267 gene polymorphisms identified in this study were not present in the ISBT database. The highly complex ABO/RH(D) and MNS systems were also identified by WGS. Blood group typing with WGS is feasible but requires improvements in reading depth for precision typing of single nucleotide variant polymorphisms (123). An automated RBC typing algorithm based on WGS data was 100 % concordant with serological methods for ABO and D antigens and 99.5% accurate at typing the C antigen (124). In the future, WGS may play an essential role in detecting rare blood types and RBC diseases.

Although genetic testing of blood types shows a decisive ascendancy, it has certain limitations. First, genetic testing requires highly trained technicians and hardware support, and some blood transfusion departments may have to rely on reference laboratories for RBC antigen genotyping. Secondly, it is not suitable for emergency blood transfusions or patients with complex blood groups since it is time-consuming. Finally, the high cost of genetic testing also limits its clinical applications. In summary, genomics is influencing all areas of medicine. Blood group genotyping is currently being used as an alternative and supplementary tool to serological testing to determine blood types for the donor-recipient match in safe blood transfusion (125).

## Rare Blood Groups Testing

A rare blood group is defined by the AABB as one with a frequency of <1/1,000 (4). Rare blood types may cause hemolytic reactions or other adverse effects after transfusion, as well as neonatal hemolytic disease. Both serological and genetic tests are available for detecting rare blood groups. Serological detection depends on the visible agglutination of RBCs in the presence of specific antibodies. However, most rare blood group antibodies are of the IgG subtype. Due to the low molecular weight of IgG antibodies and the short distance between two antigen-binding fragments, they cannot overcome the electrostatic force on the surface of RBCs. The surface negative charge on the RBCs needs to be reduced to allow visible agglutination. Therefore, rare blood group antibodies generally cannot be detected in saline media. The enzyme treatment method, the polybrene method, or the microcolumn gel method may be necessary (126). Generally, it can also increase the sensitivity of antigen and antibody and the reaction speed by adding low-ion medium and polyethylene glycol reagents, greatly shortening the detection time and making the results easy to interpret.

Indeed, pre-transfusion compatibility testing largely relies on serological testing, but antibody reagents are not available for a large number of rare blood group antigens testing with regard to rare blood requirements. Genotyping overcomes serology limitations, has the capacity of high-throughput testing, and allows easier detection of rare blood group antigens. As already discussed, PCR-RFLP, PCR-SSCP, DNA sequencing, etc., are the common genetic tests used for blood typing. They can be used to identify rare blood types and study the inheritance and frequency of rare blood antigen genes, such as real-time fluorescent PCR (95, 96). NGS is also suitable for identifying rare blood types without knowing specific antibodies (110, 111). Currently, there are more rapid and cost-effective genotyping kits with multiplex capacity and high-throughput volumes, enabling affordable large-scale rare blood group genotyping (127).

Additionally, some novel detection methods can also detect rare blood groups using small amounts of samples, such as Kleenex paper towel-based elution and direct flow-through methods (34), bromocresol green paper strip (41), nitrocellulose-based lateral-flow technique (MDmulticard^®^) (128), microfluidic thread-based analytical device (50), SPR technique (73), etc. These rare blood group detection approaches can shed light on the most promising strategies.

## Conclusions

In summary, new technologies for blood group testing are constantly emerging. Novel paper strips, biosensors, SPR, microfluidic devices, chips, gene sequencing, etc., can accurately and rapidly detect blood groups but may be hindered by prolonged turnaround and/or high costs. The classification and comparison of blood group typing methods are shown in Table 2. Emerging testing techniques have certain limitations and need to be verified further for more comprehensive applications. Currently, tube testing, microcolumn gel testing, etc. (serological methods) are still the primary methods for ABO and RH typing; these approaches are generally trustworthy and suitable for routine use. However, it also has certain drawbacks and is unable to meet the increasing demand for blood types for varieties of RBC antigens. The serological methods currently used in conjunction with evolving typing technologies can be of great value for safe blood transfusion. Furthermore, the development and validation of rapid, cost-effective, simple, and point-of-care assays for blood groups will benefit bedside pre-transfusion compatibility testing, as well as rapidly determining blood typing in emergency scenarios or remote areas where access to laboratory facilities is unavailable. It is hoped that the study of blood group typing will continue to develop and complete generations of updates and advancements.

**Table 2:** The classification and comparison of blood group testing methods.

	**Principle**	**Test method**	**Character**	**Time**	**Cost**	**Application**
Manual	Agglutination	Slide	Fast, insensitive	5–10 min	Low cost	Clinical
		Tube	Sensitive, time-consuming	10–20 min	Cost-effective and efficient	Clinical
		Novel paper-based, dye-assisted paper-based detection	Quickness, high sensitivity	<30 s	Moderate cost	Emergency
	Cell sorting	Flow cytometry	High specificity and sensitivity, quantify the antibodies	<1 hr	Not expensive	Clinical
Automated	Agglutination	Microplate	Highly sensitive	10–30 min	Moderate cost	Clinical
		Gel	Highly sensitive, time-consuming	10–45 min	Moderate cost	Clinical
	Microfluidic	Microfluidic	Fast	<6 min	Not costly	Emergency
	Magnetic	EMT	Simple and well adapted for medium- and large-sized facilities	10–30 min	Not costly	Clinical
	Fluorescence or magnetic bead	Protein chip	Sensitive, specificity, high throughput	>1 hr	Costly	Clinical
	Sensor	SPR	Sensitive	<12 min	Expensive	Research
		WM	Fast, stable, versatility, easy to control	<3.5 min	Not costly	Emergency
	Nucleic acid amplification; sequencing	Genotyping	Time-consuming, highly sensitive	>3 hrs	Costly	Clinical

## Author Contributions

KG had the idea for the article. H-YL and KG performed the search and wrote and reviewed the manuscript. All authors read and approved the final manuscript.

## Conflict of Interest

The authors declare that the research was conducted in the absence of any commercial or financial relationships that could be construed as a potential conflict of interest.

## Publisher's Note

All claims expressed in this article are solely those of the authors and do not necessarily represent those of their affiliated organizations, or those of the publisher, the editors and the reviewers. Any product that may be evaluated in this article, or claim that may be made by its manufacturer, is not guaranteed or endorsed by the publisher.

## Abbreviations

DRAM, Direct: real-time allele-specific PCR and melting curve analysis
EMT: Erythrocyte-magnetized technology
FUT: Fucose transferase
ISBT: International Society of Blood Transfusion
MALDI-TOF-MS: Matrix-assisted laser desorption/ionization time-of-flight mass spectrometry
NGS: Next-generation sequencing
PCR: Polymerase chain reaction
PCR-ASP: PCR-allele specific primer
PCR-RFLP: PCR-restriction fragment length polymorphism
PCR-SSCP: PCR-single strand conformation polymorphism
PCR-SSO: PCR-sequence specific oligonucleotide
PCR-SSP: PCR-sequence specific primer
RBC: Red blood cell
SNP: Single nucleotide polymorphism
SPR: Surface plasmon resonance
WGS: Whole-genome sequencing
WM: Waveguide-mode.
